# Supplementary material for: Development and validation of a multivariable model to identify candidates for oral cancer screening in Nigeria
Source: Commun Med (Lond). 2025 Nov 19;5:480. doi: 10.1038/s43856-025-01178-x (PMC12630950; doi:10.1038/s43856-025-01178-x)
Supplement: Supplementary file 2 — Supplementary Information [file 43856_2025_1178_MOESM2_ESM.pdf]

Table S1: Comparison of supervised learning models for identifying candidates for oral cancer screening.

Models	Cross-validation AUC Mean(SD)	Test AUC (95%CI)	Test Brier Score (95%CI) (Before Platt Scaling)
Logistic regression	0.61 (0.14)	**0.74 (0.72-0.76)**	0.20 (0.18-0.22)
Random forest	0.65 (0.11)	0.67 (0.64-0.70)	0.22 (0.20-0.24)
Gradient Boosting	0.62 (0.13)	0.69 (0.67-0.72)	**0.06 (0.05-0.07)**
Adaptive Boosting	0.60 (0.10)	0.63 (0.60-0.66)	0.24 (0.22-0.26)
Extremely randomized trees	0.63 (0.10)	0.69 (0.67-0.72)	0.22 (0.20-0.24)

AUC: Area under the receiver operating characteristic curve

SD: Standard deviation

Values in bold represent the best performance

Table S2: Comparison of different class imbalance correction techniques for identifying candidates for oral cancer screening.

Models	SMOTE		ADASYN	
	Test AUC	Test Brier Score (Before Platt Scaling)	Test AUC	Test Brier Score (Before Platt Scaling)
Logistic regression	0.47	0.27	0.44	0.27
Random forest	0.38	0.26	0.40	0.26
Gradient Boosting	0.38	0.27	0.36	0.26
Adaptive Boosting	0.46	0.25	0.46	0.25
Extremely randomized trees	0.58	0.25	0.53	0.25

AUC: Area under the receiver operating characteristic curve
SMOTE: Synthetic minority oversampling technique
ADASYN: Adaptive synthetic technique

Table S3: Stratification of model AUC and Brier scores at external testing by demographic characteristics and risk habit

Demographic characteristics and risk habits (n)		Test AUC	Test Brier Score (after Platt scaling)
Sex	Female (647)	0.62	0.02
	Male (654)	0.79	0.04
Smoking	Non-smoker (1129)	0.66	0.02
	Ever-smoker (172)	0.68	0.08
Tobacco snuff	Non-user (1268)	0.72	0.03
	Ever-user (33)	0.85	0.10
Alcohol	Non-drinker (722)	0.67	0.02
	Ever-drinker (579)	0.72	0.04

AUC: Area under the receiver operating characteristic curve
